# Mirabegron, a Selective β3-Adrenergic Receptor Agonist, as a Potential Anti-Obesity Drug

**DOI:** 10.3390/jcm12216897

**Published:** 2023-11-02

**Authors:** Anna Maria Dąbrowska, Jarosław Dudka

**Affiliations:** 1 Department of Toxicology, Medical University of Lublin, Jaczewskiego Street 8b, 20-090 Lublin, Poland; jaroslaw.dudka@umlub.pl; 2 Endocrinology Outpatient Clinic, Lublin, Poland

**Keywords:** obesity, β3-adrenergic receptor agonist, mirabegron, brown adipose tissue, “beige” adipose tissue

## Abstract

Obesity is becoming a global health epidemic. Brown and “beige” adipose tissue may produce heat, leading to energy expenditure enhancement and weight loss. Mirabegron, a selective β3-adrenergic receptor agonist, has been found to be effective as a brown adipose tissue activator, a “beige” cells stimulator and a metabolic homeostasis controller in animal and human studies. Although in animal studies, administration of mirabegron led to obesity improvement, significant weight loss in obese patients after mirabegron treatment has not been demonstrated so far, which may be associated with the too-short duration of the trials and the small number of participants in the studies. In humans, the most effective treatment for adipose tissue stimulation was high doses of mirabegron; however, cardiovascular side effects may limit the use of such doses, so the long-term safety must be evaluated. In cases of tachycardia or blood pressure elevation, the co-administration of a β1-adrenergic receptor blocker may be useful. It should be checked whether smaller doses of mirabegron, taken for a longer time, will be sufficient to stimulate brown and “beige” adipose tissue, leading to weight loss. The introduction of mirabegron into obesity treatment in the future will require long-term trials with larger numbers of subjects, to assess mirabegron efficacy, tolerability, and safety.

## 1. Introduction

Obesity is a metabolic disease that results when long-term energy intake exceeds energy expenditure [1]. It is a chronic condition that leads to numerous other serious diseases, including type 2 diabetes, cardiovascular diseases, and some types of cancer, and consequently creates a huge health care cost burden [2,3]. Nowadays, obesity affects 35.0% of adult men and 40.4% of adult women in the United States [4]. It has been estimated that, by 2030, 86% of adult people will be overweight or obese in the U.S. and approximately 1.12 billion individuals will have obesity worldwide. The data mentioned above indicates that obesity will become a global health epidemic [2,5].

The cornerstones of weight management are lifestyle intervention with the additional supportive role of anti-obesity drugs and bariatric procedures. Currently, the available drugs approved for obesity treatment affect energy balance by reducing food intake and food reward behavior in the central nervous system (e.g., suppressing appetite) or by reducing fat absorption in the intestine. No drug with a direct effect on the increase in energy expenditure through an influence on adipose tissue has been available so far [4,6].

In humans, there are two kinds of adipose tissue with distinct physiologic functions: white adipose tissue (WAT), which is specialized for the storage of excessive triglycerides when energy intake exceeds energy expenditure, and brown adipose tissue (BAT)—with the related “beige”/“brite” adipocytes (derived from WAT)—which plays a central role in metabolizing glucose, fatty acids, and other chemicals to produce heat through activation of the thermogenic tissue-specific uncoupling protein 1 (UCP1) [4,7].

Some data suggest that BAT may be functional in adult humans [1]. β3-adrenergic receptors (ARs) are expressed not only on the urinary bladder, but also on the surfaces of brown and white adipocytes [1]. Brown and “beige” adipose tissues, containing thermogenic fat cells, can be activated by β3-adrenergic receptor (β3-AR) agonists [8]. It has been reported that mirabegron is a selective human β3-AR agonist that may stimulate BAT as well as the browning process of adipocytes derived from WAT [9,10]. The fact that activation of BAT and “beige” adipocytes can increase energy expenditure makes brown and “beige” adipose tissues the promising new targets for obesity treatment [4,11].

## 2. The Role of Adipose Tissue in the Thermogenesis and Metabolic Processes Associated with Obesity

In humans, two main types of adipose tissue, which perform different functions, exist: white adipose tissue and brown adipose tissue. Besides WAT and BAT, “brite” adipose cells, termed as “beige” adipose cells, have also been distinguished. They are derived from WAT, but their metabolic function is similar to BAT [4,7].

WAT is responsible for storing energy in the form of triglycerides, releasing lipids and serving as an endocrine gland, secreting adipokines, such as adiponectin and leptin to promote metabolic homeostasis [9,12]. In obesity, white adipocytes become hypertrophied, followed by fibrosis, adipocyte necrosis, and immune cell infiltration, which leads to local and systemic inflammation, insulin resistance, and metabolic dysfunction [9].

BAT was described for the first time in outdoor-worked Finns, in 1981, who had been exposed to low environmental temperatures [6]. Metabolically active BAT has been identified in adults via PET/CT imaging focused mainly on the supraclavicular fossa, subclavian area, and axilla, followed by the mediastinal, paraspinal, perinephric, and supradrenal areas [10,12]. Although BAT is present in humans, it becomes much less prevalent with older age and in overweight or obese people compared to lean subjects [6,9,13,14]. The missing links in obesity treatment are the drugs that may increase an amount or an activity of BAT. It has been reported that BAT volume may be increased after bariatric surgery [12]. BAT is the principal thermogenic organ in mammals, with the purpose of increasing energy expenditure in response to cold or other sympathetic nerve stimulation by releasing noradrenaline from nerve terminals to activate β3-adrenergic receptors through the process termed as nonshivering thermogenesis [2,10,11,12,13]. BAT thermogenic capacity has been estimated to be approximately 500 W/kg [6]. The adipocytes in BAT are enriched with mitochondria (their levels are higher than those in WAT), in which uncoupling protein 1 (UCP1) is highly expressed. UCP1 dissipates excess energy as heat in a process known as thermogenesis [2,15]. Adrenergic activation of lipolysis stimulates the thermogenic activity of UCP1 [2,10]. Activating UCP1 on the inner mitochondrial membrane uncouples mitochondrial respiration by uncoupling electron transport from ATP production to oxidize substrate and generate heat [4,8,16]. Long chain fatty acids, generated from the intracellular lipid pools, are transported to the mitochondria through carnitine palmitoyltransferase 1 (CPT1) and used as a fuel source by brown adipocytes to produce heat. Additionally, free fatty acids have been proposed to act as allosteric activators of UCP1. In addition to fatty acids, circulating glucose can also be used by active BAT to fuel thermogenesis [2,10,17]. To sum up, BAT consumes glucose and lipids to generate heat by uncoupled respiration mediated by UCP1, leading to improved glucose and lipid homeostasis [9,13,18]. 

Human thermogenic adipocytes can originate from two distinct lineages, not only from constitutive brown adipocytes but also from recruitable “beige” cells termed as “brite” adipocytes or “brown-like” adipocytes [5]. “Beige” adipocytes are located predominately in WAT depots [16]. Adipose cells from WAT can be converted into thermogenic “beige” adipocytes in a process called “browning” or “beiging” [12]. On the one hand, substantial “beiging” of human subcutaneous WAT has been demonstrated with some disorders, such as cancer cachexia, burns, and conditions with high catecholamine levels, e.g., pheochromocytoma [8,13,14]. Patients with catecholamine-secreting tumors have also more brown adipose tissue than most people [19]. On the other hand, “brite” adipocytes can be activated with the induction of UCP1-expressing by environmental stimuli, such as cold exposure and β-adrenergic agonists mediated by the p38-MAPK signaling pathway [9,12,14,16]. The “beiging” response of obese subjects to cold is similar to lean subjects [14]. Although these cells differ from conventional brown adipocytes—as they develop from a white adipocyte precursor cell and not a brown adipocyte precursor cell, similar to classic brown adipocytes in BAT—“beige” adipocytes possess multilocular lipid droplets, a large number of mitochondria and unique brown fat gene expression markers, such as UCP1, increasing the tissue capacity for fuel oxidation and energy expenditure [4,16,18]. Moreover, it has been proven that “beiging” is associated with reduced adipose tissue fibrosis and adipose dysfunction. These findings suggest that inducing “beige” adipose tissue may improve metabolic homeostasis by increasing the ability of subcutaneous WAT to function as a metabolic sink for glucose and lipids or by reducing the WAT dysfunction that occurs with obesity [13]. Thus, in addition to thermogenesis and energy expenditure, brown and “beige” adipose tissues are associated with improved glucose and lipid homeostasis, as well as with improved insulin sensitivity in humans and mice [14]. 

Considering the fact that in adult obese people there is less BAT than in lean subjects, excess WAT, which may be stimulating and undergoing the process of “beiging”, may play the additional role apart from BAT in metabolic processes [13]. Therefore, brown adipose tissue and “beige” adipose tissue have been recognized as critical regulators of whole-body metabolism and energy expenditure and are considered promising targets for anti-obesity therapeutics [2,12,15].

## 3. The Brown, the “Beige” Fat, and the β3-Adrenoceptors in the Context of Obesity

The human β-adrenergic receptor (AR) family consists of β1, β2, and β3 receptors, in which the β1-AR is highly expressed throughout the cardiovascular system, the β2-AR is found in the pulmonary airways, throughout the vasculature, and in skeletal muscle, and the β3-AR expression is restricted mostly to the urinary bladder and the gallbladder, as well as BAT and WAT [4,10]. The human β3-AR, identified in 1989, is a 7-transmembrane receptor, with an extracellular N-terminal tail and an intracellular C-terminal tail, comprising 408 amino acids. It primarily couples to Gs to activate adenylate cyclase, resulting in increased intracellular levels of cAMP, although promiscuous coupling to other effectors, such as Gi, has been reported [4,16].

β3-AR plays a critical role in the adipose tissue, in the regulation of thermogenesis, glycolysis, and lipolysis [16]. Animal studies have shown that chronic stimulation of BAT leads to improved glucose tolerance and insulin sensitivity and reduced obesity, as well as to the release of adipokines that beneficially regulate metabolism [1,8,10,12,20]. What is more, β3-AR-mediated activation of WAT can increase pancreatic β cell insulin secretion [5]. It has also been reported that a significant proportion of nonshivering thermogenesis takes place in brown adipose tissue and is mediated primarily by the β3-adrenoceptor [19]. In chow- and high-fat diet-fed mice, BAT transplantation decreased body weight, increased glucose metabolism and insulin sensitivity, and increased glucose uptake into BAT and WAT [15].

Besides the metabolic function of β3-AR, it demonstrates roles in the brain—being involved in the processes of memory, learning, and regulation of appetite—in the gastrointestinal tract, where it takes part in the regulation of motility, and in the genitourinary system, where it plays a role in the regulation of bladder function [16].

Throughout the thermogenesis, β3-adrenoceptors increase the energy expenditure, which may lead to fat loss, in response to sympathetic stimulation [19]. There is evidence that chronic stimulation of sympathetic nervous activity and β3-ARs may activate BAT [6]. It has been proven that cold exposure stimulates the sympathetic nervous system to release noradrenaline from sympathetic nerve endings to activate β-ARs on BAT cell membranes, promoting thermogenesis. In this way, human BAT is found to start thermogenesis through the consumption of fatty acids and glucose and, further, to generate heat [1,3,8]. Moreover, β3-ARs activation by cold exposure or pharmacological agents induces a “beiging” program in WAT [18]. One way to increase the effective amount of brown adipose tissue may be to administer the β3-adrenoceptor agonist chronically [19]. A single dose of β3-adrenoceptor agonist can at least double energy expenditure in a mouse model at about 21 °C [19].

The role of the β3-ARs in human energy metabolism is supported by clinical trials, reporting associations between specific polymorphisms in the human *ADRB3* gene (the gene encoding the β3-AR) and higher rates of obesity, insulin resistance, and diabetes [10]. Moreover, mutations in the gene *ADRB3* have been correlated with insulin resistance, increased risk of obesity and diabetes, and nonalcoholic fatty liver disease in obese individuals [10]. Data indicate that the silencing of *ADRB3* in human “brown”/“beige” adipocytes alters the cellular thermogenic machinery and causes a reduction in the expression levels of genes associated with fatty acid metabolism, mitochondrial mass, and thermogenesis without compromising the “brown”/”beige” phenotype [10].

## 4. Mirabegron as a β3-AR Agonist

Mirabegron is a new generation of β3-adrenoceptor agonists with good bioavailability [21]. The effects of the selective β3-AR agonist mirabegron on bladder relaxation were discovered in 2007. For the first time, the β3-selectivity of mirabegron (YM-178) in the context of bladder function was described by Takasu et al. [22]. YM-178 increased cyclic AMP accumulation in Chinese hamster ovary cells expressing the human β3-adrenoceptor. Mirabegron demonstrated nanomolar EC50 values against the human β3-AR in biochemical assays, with potent selectivity over the β1- and β2-ARs [22]. In vivo studies demonstrated that the administration of mirabegron reduced intravesicular pressure and spontaneous bladder contractions in a dose-dependent manner [23]. Mirabegron was approved by the U.S. Food and Drug Administration (FDA) in 2012 as a new type of pharmacological treatment for overactive bladder (OAB) [6,21,24]. Fifty milligrams of mirabegron is the dose recommended to all OAB patients [24]. The drug is generally well-tolerated, with the most common side effects including hypertension, nasopharyngitis, and urinary tract infection [6].

Mirabegron β3-selectivity has been confirmed in many studies with the use of cell lines expressing both animal and human β3-adrenoceptor [22,23,25]. Mirabegron showed more than 400-fold greater selectivity for human β3-AR over human β1-AR or β2-AR [26]. For example, Brucker et al. [27] used Chinese hamster ovary (CHO)-K1, human embryonic kidney 293 cells stably expressing human β1-, β2-, or β3-adrenergic receptors, and human α1D- and α2B-adrenergic receptors to assess mirabegron selectivity. At a concentration of 10 μM, β3-adrenergic activity relative to isoproterenol (full β-adrenergic agonist) was 88% for mirabegron. In turn, β1- and β2-adrenergic activity for mirabegron was 3% and 15%, respectively [27]. Mirabegron did not meet the significance criterion for the inhibition of α1D- or α2B-adrenergic receptors in this study [27]. However, some studies indicated that mirabegron may play a role as an α1-adrenoceptor antagonist [28,29]. Alexandre et al. [28] postulated that mirabegron relaxed urethral smooth muscle in mice by a dual mechanism involving β3-adrenoceptor activation and α1-adrenoceptor blockade. In another study, mirabegron induced endothelium-independent vasorelaxation in arteries from visceral adipose tissue through the antagonism of α1-adrenoceptors. This action suggested that mirabegron might effectively improve visceral adipose tissue perfusion, thereby favoring healthy adipose tissue remodeling and preventing some of the unwanted cardiometabolic consequences of obesity and aging [29]. It still remains difficult to determine to what extent α1-adrenoceptor antagonism may contribute to the clinical effects of mirabegron [28,29].

The beneficial metabolic changes caused by chronic mirabegron treatment may come from stimulation of the β3-AR on human BAT and WAT [5,12,18]. It has been suggested that mirabegron could improve obesity-related metabolic disease by increasing BAT thermogenesis, WAT lipolysis, and stimulation of the “browning” process of adipocytes derived from WAT [4,5,9,10]. Acute mirabegron treatment enhanced energy expenditure [10,15]. After silencing β3-AR expression, mirabegron could not stimulate BAT lipolysis and thermogenesis [10].

Many studies showed that mirabegron treatment increased glucose uptake into brown and “beige” adipocytes, improved glucose homeostasis, and increased both insulin sensitivity and β cell function [1,9]. Moreover, it has been shown that chronic treatment with β3-AR agonists in humans may release beneficial adipokines [1]. The way mirabegron improves glucose metabolism has not been clarified so far [5]. However, some mechanisms have been postulated. Firstly, mirabegron stimulates the secretion of adiponectin, which is known to be a WAT-derived adipokine associated with higher insulin sensitivity in skeletal muscle and liver. Secondly, mirabegron elevates the concentration of gastric inhibitory polypeptide (GIP), the incretin connected to insulin secretion. Finally, the mirabegron mechanism may involve the β cells themselves [5].

The β3-adrenergic receptor agonist is an excellent candidate for the treatment of obesity because the β3 isoform is expressed exclusively in adipocytes, and the action on other cell types, such as cardiomyocytes and smooth muscle cells through the other β isoforms—β1 and β2—is minimal and dose-dependent [11]. Therefore, as a β3-AR agonist, mirabegron would activate thermogenesis in adipose tissue, stimulating lipid oxidation and glucose consumption to produce heat, without causing serious cardiovascular side effects [13].

## 5. Mirabegron as an Anti-Obesity Agent in the Experimental Studies

Treatment of rodents with β3-AR agonists activated BAT, resulting in increased energy expenditure, weight loss, and improved glucose and lipid metabolism. It also restored the NO/redox balance, improved endothelial function and, thus, exerted vascular protective effects [4,6,13,17]. Increased BAT activity prevented the development and severity of obesity and type 2 diabetes, while BAT-deficient mice were obesity-prone [16]. It has been reported that a transgene-induced reduction in BAT mass in mice produced obesity, and these mice had further increased susceptibility to obesity due to obesitogenic diets [8,30,31].

As was mentioned above, mirabegron may be effective as a BAT activator, a “beige” cells stimulator, and a metabolic homeostasis controller. The beneficial influence of mirabegron on metabolism has been confirmed in both in vitro and in vivo studies [2,4,15,18].

In the study conducted by Dehvari et al. [15], the effects of mirabegron in brown, white, and “beige” adipocytes in vitro, and its effects on glucose utilization and thermogenesis in vivo, were reported. It was shown that mirabegron increased glucose uptake and glycolysis in mouse brown adipocytes in vitro and promoted glucose uptake into BAT in vivo. It increased cAMP levels and UCP1 mRNA, resulting in increased UCP1-mediated oxygen consumption, as well as glucose uptake and cellular glycolysis in brown and “beige” adipocytes (there was a lack of such action in the primary cell cultures of brown adipocytes from β3-adrenoceptor knockout mice), and these effects were either absent or reduced in white adipocytes. In vivo, mirabegron increased whole body oxygen consumption and glucose uptake into brown and inguinal white adipose tissue and improved glucose tolerance. In β3-adrenoceptor knockout mice, mirabegron failed to induce glucose uptake into adipose tissue, as well as to increase whole body oxygen consumption, which proves that β3-adrenoreceptor signaling is a main pathway of mirabegron metabolic actions [15].

Similar to Dehvari et al. [15], Hao et al. [4] investigated the anti-obesity effects of mirabegron using in vitro and in vivo models. In both—mouse brown preadipocytes cell line and 3T3-L1 white preadipocytes—mirabegron stimulated UCP1 expression. Mirabegron-treated mice, fed a high-fat diet, had lower body weight and adiposity, as well as improved glucose tolerance and insulin sensitivity. Lipid droplets in BAT of mirabegron-treated mice were fewer and smaller in size compared to controls. H&E staining and immunohistochemistry indicated that mirabegron increased the abundance of “beige” cells in WAT [4]. It was concluded that mirabegron enhanced UCP1 expression and promoted “browning” of WAT, and these were accompanied by improved glucose tolerance, insulin sensitivity, and the prevention of high-fat diet-induced obesity [4]. In another animal study, Valgas da Silva et al. [18] reported that 2-week treatment with mirabegron decreased inflammation, improved metabolism, prevented BAT and liver ectopic fat accumulation, and decreased insulin resistance in obese mice (decreased HOMA index and insulin levels). Mirabegron increased UCP1 expression in BAT and the energy expenditure and also decreased adiposity in obese mice. What is more, mirabegron decreased circulating levels of free fatty acids, glycerol, and TNF-α. It is known that increased circulating FFA levels cause insulin resistance in insulin target organs and have emerged as a major link between obesity and the development of metabolic syndrome. It is also known that TNF-α has a lipolytic effect, which results in increased levels of FFA and glycerol in the circulation, contributing to insulin resistance. However, in contrast to the study conducted by Dehvari et al., no changes in inguinal WAT were found—mirabegron did not induce “beiging” of inguinal WAT from the obese mice. Moreover, diet-induced obesity significantly increased the lipid deposits in the liver and BAT, but mirabegron partially reversed those changes, which may indicate a protective role of mirabegron in the development of hepatic steatosis and insulin resistance [18].

The confirmation that mirabegron may be useful as an anti-obesity agent was also found in the Hao et al. study [4]. It was proved that mirabegron causes a 14-fold increasing in gene expression of UCP1 and may result in a 12% weight loss and a reduction of adiposity in obese mice compared to physical activity.

The combined therapy, composed of mirabegron and metformin, was checked in the prevention mouse model, as well as in the treatment mouse model of obesity [2]. Metformin, a derivative of biguanide, is one of the drugs most commonly used to treat type 2 diabetes. It inhibits mitochondrial complex I, vital to electron transport, which leads to AMPK (adenosine 5′-monophosphate-activated protein kinase) activation. As a result, the production of ATP (adenosine triphosphate) decreases, and the intracellular concentration of ADP (adenosine diphosphate) increases. Consequently, the cellular levels of AMP (adenosine monophosphate) increase, finally activating AMPK. AMPK is a key regulator of numerous metabolic pathways, including glucose and lipid metabolism and energy homeostasis. Metformin also plays important roles by inhibiting insulin and IGF receptor signaling, resulting in changes in metabolic homeostasis [32]. Zhao et al. [2] indicated that this complex therapy could be a promising approach for the prevention and treatment of obesity, by targeting both energy intake and energy expenditure simultaneously, with no side effects on cardiovascular function. In the prevention model, metformin and mirabegron caused further 12% and 14% reductions in body weight gain induced by a high-fat diet, compared to metformin or mirabegron alone, respectively. In the treatment model, metformin and mirabegron additively promoted 17% body weight loss in diet-induced obese mice, which was 13% and 6% greater than metformin and mirabegron alone, respectively. The combined therapy had an additive effect on weight loss in mice, which was associated with significant fat loss, especially in subcutaneous WAT [2]. The researchers suggested that the additive effect of metformin and mirabegron on enhancing energy expenditure was a major contributor to reduced body weight and fat mass in the mice [2]. Metformin and mirabegron therapy had an additive effect on BAT thermogenesis and subcutaneous WAT browning. The combined therapy significantly upregulated UCP1 expression in BAT and subcutaneous WAT [2]. Additionally, metformin and mirabegron improved glucose tolerance and insulin sensitivity, and the effect was independent of food intake. However, coadministration of metformin and mirabegron did not improve glucose homeostasis in mice any more than metformin or mirabegron alone [2].

## 6. Mirabegron as an Anti-Obesity Drug—The Data from the Human Studies

Besides experimental studies, there are many clinical trials in which the influence of mirabegron on BAT activity and body mass has been shown. The authors reported that mirabegron led to increased BAT activity and resting energy expenditure [1,3,5,10,17,21]. Preliminary evidence suggests that the effects of mirabegron on glucose metabolism, HDL cholesterol, and bile acids resemble those achieved through mild exercise [1,5].

In the first group of studies, mainly high doses of mirabegron (100 mg, 150 mg or 200 mg) were tested [1,3,5,17,21].

Cypess et al. [1] used, for the first time, mirabegron to study human BAT and compared its action to a degree that matched responses to cold exposure. The administration of 200 mg per day of oral mirabegron for 12 weeks to 12 healthy men was associated with higher BAT activity (measured via 18F-fluorodeoxyglucose positron emission tomography combined with computed tomography) and the elevation of the resting metabolic rate by 203 ± 40 kcal/day, compared to individuals receiving the placebo. It was posited that the calculated weight loss, associated with energy expenditure, should reach 5 kg in the first year and 10 kg by the end of 3 years [1]. In this study, the 200 mg dosage of mirabegron, a much higher dose than those currently approved for reducing the symptoms of overactive bladder, was generally well-tolerated, even after 12 weeks of daily oral administration [1]. The most common side effect was tachycardia [1].

The high dose of mirabegron [100 mg per day] was tested by O’Mara et al. during a 4-week therapy program in 14 healthy women of various ethnicities [5]. In the primary endpoint, the researchers reported that chronic mirabegron therapy increased BAT volume and metabolic activity, measured by 18F-fluorodeoxyglucose PET/CT [5]. What is more, women who had had primarily less BAT finally reached a larger increase in BAT volume and activity after treatment [5]. The secondary endpoints revealed that whole-body resting energy expenditure was higher after treatment of mirabegron; however, no changes in body weight or composition were found. These findings should be associated with a narrow BMI range and participation of non-obese women. Moreover, mirabegron therapy was found to elevate lipoprotein biomarkers such as HDL and apolipoprotein A1, apolipoprotein E, and gastric inhibitory peptide (GIP), as well as adiponectin levels—anti-diabetic and anti-inflammatory adipokine. After mirabegron treatment, a reduction in the ApoB100/ApoA1 ratio, a biomarker of cardiovascular risk, was observed. Finally, after chronic mirabegron treatment, an intravenous glucose tolerance test revealed higher insulin sensitivity, glucose effectiveness, and insulin secretion [5]. However, the change in the homeostatic model assessment of insulin resistance (HOMA-IR), a measure of insulin resistance, was not significant after chronic mirabegron treatment. The authors suggested that the main reason should be the near-normal HOMA-IR level at study initiation [5]. As is a concern common in chronic treatment with adrenergic agonists, 100 mg of mirabegron led to a diurnal variation in heart rate such that mirabegron increased it more overnight than when the subjects were awake and moving. On the other hand, mirabegron treatment had no effect on exercise tolerance [5].

Loh et al. [21] reported the efficacy of various single doses of mirabegron (50, 100, 150, and 200 mg) in a group of 17 healthy individuals (11 men, 6 women) who took the drug on four separate days, with 3 to 14 days of wash-out between each dose. They reported that energy expenditure (measured by indirect calorimetry) significantly increased after the 100 mg and 200 mg doses and trended towards an increase after the 150 mg doses but was not significantly different from baseline in response to 50 mg of mirabegron. Supraclavicular skin temperature (as a surrogate indicator of BAT activity), increased after the 50 mg, 100 mg, and 150 mg mirabegron doses, but was not significantly different from baseline in response to 200 mg. Considering the side effects, the change in systolic blood pressure was significant after the 150 mg and 200 mg doses compared to the 50 mg dose and the 100 mg dose. However, there was no difference in diastolic blood pressure between the 50 mg, 100 mg, 150 mg, and 200 mg doses. The change in heart rate was greater after 200 mg compared with remaining doses. They concluded that a 100 mg dose of mirabegron may be efficacious to increase energy expenditure and supraclavicular skin temperature in a β3-adrenoceptor-specific manner, without the significant elevations in blood pressure or heart rate observed at higher doses [21].

Baskin et al. [17] studied the clinical implications of mirabegron in 12 lean, healthy men administered the approved dose of 50 mg as well as a high dose of 200 mg. There was a more-than-dose-proportional increase in BAT metabolic activity (measured by PET/CT). Compared with the placebo, 50 mg of mirabegron increased BAT activity in most subjects. However, BAT activation with 50 mg was significantly less than with 200 mg. Only the 200 mg dose elevated resting energy expenditure (5.8%). The cardiovascular stimulation was consistent with previous studies, as 200 mg of mirabegron increased both heart rate and blood pressure.

A randomized, double-blinded, cross-over study consisting of three interventions—short-term (~2 h) cold exposure, mirabegron (200 mg single dose), and placebo—in a group of 10 lean Dutch South Asian and 10 lean Europid men, conducted by Nahon et al. [3], revealed that cold exposure and mirabegron induced beneficial metabolic effects, including an increase in resting energy expenditure (measured by indirect calorimetry), serum free fatty acids levels, and lipid oxidation. Mirabegron increased heart rate both in South Asians (+10 beats/min) and white Caucasians (+7 beats/min), while systolic and diastolic blood pressure were not significantly changed [3]. It was observed that a single dose of mirabegron increased serum insulin levels without affecting glucose levels. Mirabegron may stimulate insulin release directly through acting on the β3-AR on the pancreas or indirectly through an increase in FFA that may stimulate the pancreas to release insulin [3].

The dose-dependent action of mirabegron on adipose tissue, including the influence on BAT activity and energy expenditure, may be analogous to the effect of mirabegron on the urinary bladder. Activation of β3-adrenergic receptors with mirabegron resulted in concentration-dependent β3-adrenergic receptor responses [27]. Regarding bladder function, in in vivo studies, the administration of mirabegron reduced intravesicular pressure and spontaneous bladder contractions in a dose-dependent manner [23].

It was reported that high doses of mirabegron (especially 200 mg per day), much higher than those approved by the FDA for bladder overactivity (50 mg per day), may be associated with cardiovascular side effects such as headaches, tachycardia, and elevated blood pressure (mostly only systolic blood pressure) [1,3,5,17,21]. The increase in systolic blood pressure may reach ~10 mm Hg at the dose of 200 mg daily [21]. This is the result of loss of selectivity for the β3-adrenoceptor at this dose, such that mirabegron indirectly activates β1-adrenoceptors that are widely expressed throughout various organs, particularly the cardiovascular system. This mechanism involves noradrenaline transporter uptake of mirabegron into cardiac sympathetic nerve terminals, subsequently causing a release of noradrenaline, which activates β1-adrenoceptors [21]. However, mirabegron treatment had no effect on exercise tolerance [5]. Activation of β1-adrenoceptors may be attenuated by co-administration of either propranolol or bisoprolol [16]. On the other hand, clinical trials revealed that mirabegron doses up to 100 mg per day for at least 12 months showed a good safety profile and did not result in increased incidence of tachycardia, blood pressure, ECG changes, or any cardiovascular events [21]. Lower therapeutic doses (50 mg) in OAB patients resulted in minor changes in pulse rate (1 beat per minute) and blood pressure (1 mm Hg or less). Considering cardiovascular side effects, mirabegron is not recommended in patients with severe uncontrolled hypertension (systolic blood pressure ≥ 180 mm Hg and/or diastolic blood pressure ≥ 110 mm Hg) [16].

In the second group of studies, conducted by Finlin et al. [9,13,14], a low dose of mirabegron, one that has been approved for OAB treatment, was tested.

In a group of 13 middle-aged obese patients, 50 mg of mirabegron per day during a 12-week therapy induced “beiging” of subcutaneous WAT, as well as improved β-cell function. Mirabegron increased protein expression of the “beige” adipose markers UCP1 (2.4-fold), transmembrane protein 26 (TMEM26) (4.2-fold), and cell death-inducing DFFA-like effector A (CIDEA) (2.4-fold) [13]. The “beiging” of subcutaneous WAT by mirabegron may reduce adipose tissue dysfunction, which may enhance muscle oxidative capacity and may improve β cell function [13]. Taking into consideration glucose homeostasis, mirabegron treatment improved oral glucose tolerance, leading to convert prediabetes into normal glucose concentration, reduced hemoglobin A1c levels, and improved insulin sensitivity and β cell function, without affecting fasting blood glucose or fasting insulin levels and HOMA-IR. However, the results from euglycemic clamps, which are the gold standard for measuring insulin sensitivity, revealed that mirabegron treatment consistently and significantly increased the glucose infusion rate by approximately 12% [13]. Plasma lipid levels were changed significantly, but, after mirabegron treatment, a trend toward a reduction in total cholesterol was found [13]. Unfortunately, a 12-week therapy did not result in a significant increase in amount of BAT and resting energy expenditure, weight loss, or changes in body composition in such patients [13].

The beneficial effect of mirabegron, similar to the effect of cold exposure, on the induction of “beiging” adipose tissue in human subcutaneous WAT was also reported in another study conducted by Finlin et al. [14]. They exposed lean and obese research participants to cold or treated them with mirabegron. Chronic mirabegron treatment (10 weeks; 50 mg/day) induced UCP1 (3-fold) and TMEM26 (8.7-fold) in obese subjects. What is more, the expression of UCP1 and “beige” adipocyte markers increased more than after 10 days of repeated cold exposure [14].

In the next study, composed of 12 obese insulin-resistant participants, Finlin et al. [9] evaluated the ability of pioglitazone (30 mg/day) treatment or mirabegron (50 mg/day) treatment in monotherapy, as well as a combination of pioglitazone (30 mg/day) and mirabegron (50 mg/day) treatment, to increase “beige” fat or further improve glucose metabolism during 12 weeks of therapy. Pioglitazone is a PPARγ activator that may stimulate BAT or “beige fat”. Treatment with pioglitazone or the combination of pioglitazone and mirabegron increased “beige” adipose tissue protein marker expression and improved insulin sensitivity (measured by euglycemic clamp, more effective in combined therapy) and glucose homeostasis (including improved glucose tolerance tests, more effective in combined therapy), but neither treatment induced BAT or affected energy expenditure in obese subjects. Moreover, there was no significant change in body weight after treatment. Despite the fact that mirabegron and pioglitazone administered separately induced adipose tissue “beiging”, the addition of pioglitazone to mirabegron did not enhance “beiging”, as the combination treatment resulted in less “beiging” than either drug alone [9].

Although the preliminary findings from animal studies showed the benefits from the co-administration of mirabegron and metformin in the prevention as well as in the treatment of obesity [2], to our knowledge, the influence of such combined therapy has not been checked in relation to BAT activity, energy expenditure, and weight loss in humans. It is only known that there are no clinically significant interactions between metformin and mirabegron. In the study with 32 healthy male subjects (BMI: 18–30 kg/m^2^), mirabegron (160 mg administered once daily) showed no effect on the pharmacokinetics of metformin (500 mg administered twice daily). Co-administration of mirabegron with metformin resulted in small changes in mirabegron exposure (AUC and Cmax decreased by 21%). The observed pharmacokinetic changes were not considered clinically relevant. Therefore, no dosage adjustment of mirabegron is necessary when it is co-administered with metformin [33].

Although the data confirms that a low dose of mirabegron may induce “beiging” of subcutaneous WAT, it was reported that 50 mg of mirabegron during short-term treatment (approximately 12 weeks of therapy) has no effect on the amount of BAT, resting energy expenditure, and weigh loss. Therefore, long-lasting clinical trials, with obese participants on a smaller dose of mirabegron, are needed to assess whether adipose tissue “beiging” would translate into enhancement of resting energy expenditure and meaningful weight loss.

## 7. Conclusions

Metabolically active BAT has been positively correlated to improved energy, glucose, and whole-body metabolism [34]. Activation of BAT and the induction of “browning” process in WAT seem to be an interesting therapeutic strategy to enhance energy expenditure and improve metabolism. Mirabegron, as a β3-adrenergic receptor agonist, was found to be effective as a BAT activator, a “beige” cells stimulator, and a metabolic homeostasis controller in both animal and human studies. Although, in animal studies, administration of mirabegron led to obesity improvement, significant weight loss in obese patients has not yet been demonstrated after high doses or low doses of the drug. It can be explained by the too-short duration of the trials and the small number of participants in the studies. What is more, in humans, the treatment most effective for BAT and WAT stimulation was high doses of mirabegron; however, cardiovascular side effects may limit the use of doses higher than those approved by the FDA to treat overactive bladder. On the one hand, considering the use of high doses of mirabegron, the long-term safety in relation to the cardiovascular system must be evaluated. In case of the aggravated activation of the myocardial β1-receptors, the concomitant administration of 100–200 mg of mirabegron with a β1-AR blocker may be a useful therapeutic strategy to avoid cardiovascular side effects. On the other hand, it should be evaluated whether smaller doses of mirabegron, e.g., those approved for overactive bladder (50 mg per day), taken for a longer time, will be sufficient to stimulate the BAT growth, WAT “browning”, and thermogenesis that may lead to weight loss. In clinical trials regarding the efficacy and the safety of mirabegron in patients with overactive bladder, the influence of mirabegron on body weight was not checked. To our knowledge, the efficacy of mirabegron in relation to metabolic disorders, including obesity, in subjects treated for overactive bladder, has not been assessed so far.

In our opinion, the potential role of mirabegron in the treatment or in the prevention of obesity would depend on the results of its efficacy determined by long-term trials. In the case of a lack of or an unsatisfactory weight-loss effect (compared to currently available drugs approved for obesity treatment), mirabegron could be used to improve the metabolic profile in patients with obesity. If the weight loss effect of mirabegron is confirmed, the drug will become an alternative option to current anti-obesity agents, especially in patients with contraindications or intolerance to other drugs. Moreover, an interesting aspect to assess in clinical trials would be whether the co-administration of mirabegron and other drugs, such as metformin, pioglitazone, or other currently used anti-obesity medications, could be a more effective strategy than the administration those drugs alone to improve metabolic profiles or to treat obesity. The benefits from the co-administration of mirabegron and metformin in the prevention as well as in the treatment of obesity that were proven in animal studies should be confirmed in further clinical trials. Although the preliminary findings from the co-administration of mirabegron and pioglitazone in obese participants indicated no influence of such therapy on body weight, additional studies should be performed to confirm these results. Therefore, the introduction of β3-adrenergic receptor agonists into the treatment of obesity in the future will require long-term trials with a larger number of subjects to assess their efficacy, tolerability, and safety.

Brown and “beige” adipose tissues remain an attractive target for combating metabolic disease. Further studies are needed to confirm whether the combination of BAT- and “beiging”-activating agents, physical exercises, and a healthy hypocaloric diet would be a successful strategy to achieve weight loss in patients with obesity.
